# Physiological, Agronomic, and Grain Quality Responses of Diverse Rice Genotypes to Various Irrigation Regimes under Aerobic Cultivation Conditions

**DOI:** 10.3390/life14030370

**Published:** 2024-03-12

**Authors:** Ahmed M. A. Mousa, Ahmed M. A.-G. Ali, Abdelrahman E. A. Omar, Khadiga Alharbi, Diaa Abd El-Moneim, Elsayed Mansour, Rasha S. A. Elmorsy

**Affiliations:** 1 Department of Crop Science, Faculty of Agriculture, Damietta University, Damietta 34517, Egypt; ahmedmousa@du.edu.eg (A.M.A.M.); dr_rashasaad@du.edu.eg (R.S.A.E.); 2 Department of Crop Science, Faculty of Agriculture, Zagazig University, Zagazig 44519, Egypt; ahmed_a_r_2004@du.edu.eg (A.M.A.-G.A.); omaromar1971@yahoo.com (A.E.A.O.); 3 Department of Biology, College of Science, Princess Nourah bint Abdulrahman University, P.O. Box 84428, Riyadh 11671, Saudi Arabia; kralharbi@pnu.edu.sa; 4 Department of Plant Production (Genetic Branch), Faculty of Environmental Agricultural Sciences, Arish University, El-Arish 45511, Egypt; dabdelmoniem@aru.edu.eg

**Keywords:** drought stress, arid environment, cluster analysis, principal component analysis, Mediterranean region

## Abstract

Aerobic rice cultivation represents an innovative approach to reduce water consumption and enhance water use efficiency compared to traditional transplanting methods. Simultaneously, cultivating drought-tolerant rice genotypes becomes crucial to ensure their sustainable production under abrupt climate fluctuations. Hence, this study aimed to explore the physiological, agronomic, and grain quality responses of ten diverse rice genotypes to various irrigation levels under aerobic cultivation conditions. A field experiment was performed for two summer seasons of 2019 and 2020 in an arid Mediterranean climate. The irrigation regimes were well watered (13,998 m^3^/ha), mild drought (10,446 m^3^/ha), moderate drought (7125 m^3^/ha), and severe drought (5657 m^3^/ha). The results revealed considerable variations among rice genotypes under tested irrigation regimes in all physiological, agronomic, and quality traits. According to drought response indices, rice genotypes were classified into three groups (A–C), varying from tolerant to sensitive genotypes. The identified drought-tolerant genotypes (Giza-179, Hybrid-1, Giza-178, and Line-9399) recorded higher yields and crop water productivity with reduced water usage compared to drought-sensitive genotypes. Thus, these genotypes are highly recommended for cultivation in water-scarce environments. Furthermore, their characteristics could be valuable in breeding programs to improve drought tolerance in rice, particularly under aerobic cultivation conditions. The PCA biplot, heatmap, and hierarchical clustering highlighted specific physiological parameters such as relative water content, chlorophyll content, proline content, peroxidase content, and catalase content exhibited robust associations with yield traits under water deficit conditions. These parameters offer valuable insights and could serve as rapid indicators for assessing drought tolerance in rice breeding programs in arid environments.

## 1. Introduction

Rice (*Oryza sativa* L.) is a primary cereal crop for nearly half of the global population [1]. It has a fundamental nutritional benefit with high carbohydrate content and is rich in minerals, proteins, vitamins, and calories [2]. Its global cultivated area is around 165 million hectares, producing approximately 787 million tons [3]. The cultivated area in Egypt is approximately 475 thousand hectares, with an annual production of about 5.0 million tons [3]. Rice production should be improved to ensure worldwide food security, considering the challenges of population growth and abrupt climate fluctuations [4].

The reduction in water resources is attributed to both natural factors and human-induced 45-factors and is expected to have a significant effect on water-intensive crops like rice [5,6]. Water scarcity threatens the productivity of irrigated rice ecosystems, necessitating the exploration of water-saving technologies to preserve water and sustain rice yields [7]. Aerobic rice cultivation is an innovative approach to reduce water consumption and enhance the efficiency of water use [8]. It involves directly sowing rice in non-puddled aerobic soil with supplementary irrigation and appropriate fertilization [9]. It has advantages over traditional transplanting methods, primarily in water conservation. The aerobic rice system reduces water usage by eliminating the need for standing water in lowland transplanted rice [10]. Another significant benefit of cultivating aerobic rice is preventing the loss of essential micronutrients from the soil and decreasing methane gas emissions as a product of flooded fields [11]. In this perspective, Liu et al. [8], Sandhu et al. [12], Fukai and Mitchell [13], Dey et al. [14] and Bhutto et al. [15] explained that aerobic rice cultivation represents an innovative and sustainable approach to rice farming that significantly diverges from traditional methods. It primarily addresses water scarcity in rice cultivation by promoting water efficiency and reducing the dependency on flooded conditions typically associated with paddy rice farming.

Drought stress poses a significant threat to global rice production [16,17]. The requirement for standing water throughout its growth phases makes rice vulnerable to water shortages, particularly during the reproductive stage [18]. Rice production in the Mediterranean region is substantially affected by drought stress at morphological, physiological, agronomic, and grain quality levels [19]. Water shortage significantly affects dry matter accumulation, photosynthetic capacity, and oxidative damage. Insufficient water, especially during anthesis and grain filling, considerably reduces rice productivity and quality potential by accelerating flowering, heightening spikelet sterility, and ultimately diminishing panicle traits [20]. However, rice genotypes exhibit varying capacities under water deficit conditions [21]. In this context, Kumar et al. [22], Bhandari et al. [23], Lanna et al. [24], Baldoni [25], and Ghidan and Khedr [26] demonstrated the importance of exploring the morphological, physiological, biochemical, agronomic, and quality traits of diverse rice genotypes across various irrigation regimes. Exploring these characteristics across various water conditions facilitates breeding efforts to enhance drought tolerance in rice. Consequently, this study aimed to (i) investigate physiological, agronomic, and grain quality responses of diverse rice genotypes to varying irrigation levels under aerobic cultivation conditions, (ii) identify rice genotypes with drought tolerance suitable for cultivation in arid environments, which could therefore be exploited in breeding programs for developing drought-tolerant rice varieties, and (iii) study the associations among studied morpho-physiological, agronomic, and grain quality traits under drought stress conditions.

## 2. Materials and Methods

### 2.1. Plant Material

Ten rice genotypes with a wide range of diverse genetic materials for agronomic performance were selected for the study. The chosen genotypes represent a mix of commercially adapted varieties and promising advanced lines. The evaluated plant materials included genotypes with diverse genetic backgrounds, as illustrated in Table 1. The responses of these genotypes to varying irrigation regimes were evaluated under aerobic cultivation conditions. Seeds of assessed genotypes were obtained from the Rice Research and Training Center (RRTC).

### 2.2. Experimental Site and Agricultural Practices

The field experiment was carried out over two seasons in 2019 and 2020 at the Extension Farm, Al-Husayniya, Sharqia, Egypt (30°54′ N 31°53′ E). This experimental site represents a hot climate with minimal rainfall during the rice-growing seasons (Table S1). The soil of the experimental site is clay throughout the profile (i.e., 13.4% sand, 55.9% clay, and 30.7% silt) (Table S2). The experiment was applied under aerobic conditions by directly sowing rice in non-flooded soil with supplementary irrigation. Seeds of each genotype were sown on the first of May in both seasons of 2019 and 2020 in ten rows per replication. Each row was 5 m long with spacing of 0.2 m among rows. The applied experimental design was a split plot in three replications. The irrigation treatments were randomized in the main plots, while the genotypes were distributed across the sub-plots. The genotypes were assessed under four distinct irrigation treatments; well-watered, mild drought, moderate drought, and severe drought conditions. To prevent lateral movement and maintain better water control, there were 4 m wide ditches among the main plots. The irrigation regimes were sustained from sowing until maturity, and the irrigation amounts for each treatment were quantified using a flow meter. Well-watered conditions were maintained by applying continuous flooding every four days, ensuring an adequate submersion depth to cover all surface areas with water during each irrigation cycle. The amount of water used in the well-watered treatment was 14,013 m^3^/ha in the first season and 13,984 m^3^/ha in the second season. The mild-drought treatment involved irrigation every eight days. The amount of water applied in mild-drought treatment was 10,459 m^3^/ha in the first season and 10,434 m^3^/ha in the second season. For the moderate-drought treatment, irrigation was applied every twelve days without maintaining standing water. The amount of water used in the moderate-drought treatment was 7134 m^3^/ha in the first season and 7117 m^3^/ha in the second season. The severe drought treatment received irrigation every sixteen days without maintaining standing water. The amount of water applied in the severe drought treatment was 5663 m^3^/ha in the first season and 5650 m^3^/ha in the second season. Nitrogen fertilizer was applied in three splits at a rate of 166 kg N/ha using urea (46.0% N). Additionally, phosphorus was added at a rate of 72 kg P_2_O_5_/ha as single superphosphate (15% P_2_O_5_), and potassium was applied at a rate of 54 kg K_2_O kg/ha using potassium-sulfate (48% K_2_O).

### 2.3. Measured Traits

#### 2.3.1. Physiological Parameters

Chlorophyll content was recorded at the heading stage, utilizing chlorophyll analytical apparatus (chlorophyll meter SPAD 502, Konica Minolta Inc, Tokyo, Japan). It was recorded as the amount of chlorophyll per square decimeter. Five flag leaves were used from the widest part of the leaf of the main Culm. The accumulation of proline, peroxidase, and catalase was determined following the method outlined by Bates et al. [27] and Chance and Maehly [28]. Relative water content (%) was recorded as described by Barrs and Weatherley [29]. Leaf rolling was determined as an indicator of the degree of drought tolerance on a scale of 1–9 from plant leaves. It was measured by visual estimation following the method of De Datta et al. [30], as shown in Table S3.

#### 2.3.2. Agronomic Traits

Days to heading was recorded as the number of days from the sowing date to the date of 50% panicle exertion. At harvest, rice plants of ten guarded plants were collected at random from the fifth middle row in each sub-plot to determine the following traits: number of panicles/m^2^, plant height (cm), number of filled grains panicle weight (g), and sterility percentage, 1000-grain weight (g). The grain and biological yields were determined from an area of 4 m^2^ in each plot and then converted into t/ha.

#### 2.3.3. Grain Quality Characteristics

Around 150 g of grains were collected from each treatment and mixed and sent to the laboratory of grain quality in the Rice Research and Training Center to determine grain quality characteristics following the methods of Adair [31] and Juliano [32]. Brown rice was milled by employing McGill Miller no. 2, and samples were milled for sixty seconds. Then, the milled samples were collected to record their weight and then the percentage of total milled rice was calculated as follows: $Milling\;percentage=\frac{Weight\;of\;milled\;rice\;(g)}{Weight\;of\;rough\;rice\;(g)}\times 100$. Cleaned rough rice samples with a moisture content of about 12–14 % were used to estimate the hulling percentage using an experimental huller machine (stake). $Hulling\;percentage=\frac{Weight\;of\;brown\;rice\;(g)}{Weight\;of\;rough\;rice\;(g)}\times 100$. Complete grains were split from total milled rice utilizing a rice sizing device. Then, the amount of head rice yield was obtained and calculated. Head rice percentage was calculated as follows: $Head\;rice\;percentage=\frac{Weight\;of\;whole\;milled\;rice\;(g)}{Weight\;of\;rough\;rice\;(g)}\times 100$. Broken rice percentage was calculated from rice milled, husked, or hand-pounded milled rice with grain size between 1/4 to 3/4 size of the whole grain, as follows: $Broken\;rice\;percentage=\frac{Weight\;of\;broken\;rice\;(g)}{Weight\;of\;rough\;rice\;(g)}\times 100$. The green rice percentage was calculated from rice in which only the husk had been eliminated, maintaining the bran layers and most of the germ together as follows: $Green\;rice\;percentage=\frac{Weight\;of\;green\;rice\;(g)}{Weight\;of\;rough\;rice\;(g)}\times 100$.

### 2.4. Statistical Analysis

The split-plot design was employed to analyze variance (ANOVA) across all measured traits. Variations among irrigation treatments, rice genotypes, and their interactions were detected using the least significant difference test (LSD) at a significance level of *p* ≤ 0.05. To explore their interrelationships, principal component analysis was conducted on the average values of the physiological, agronomic, and quality traits. All statistical analyses were applied utilizing R programming statistical software version 4.2.1.

## 3. Results

### 3.1. Physiological Parameters

**Figure 1:**
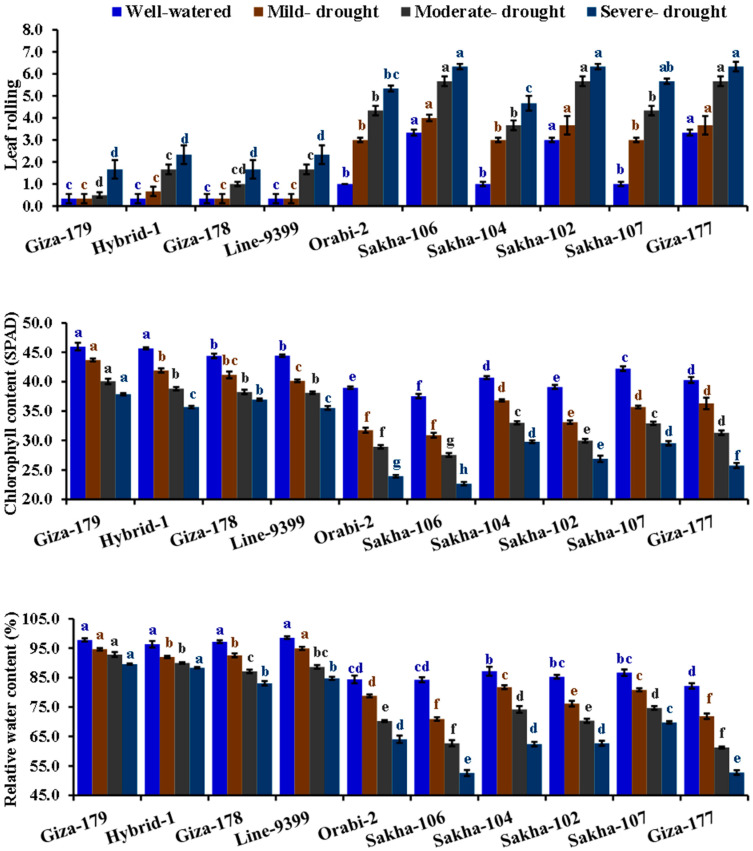
Impact of different irrigation regimes on leaf rolling, chlorophyll content, and relative water content of ten different rice genotypes over two growing seasons in 2019 and 2020. The top bars on each column indicate the standard error (SE). Distinct letters on columns of each irrigation regime (sharing the same color) reveal significant differences based on the LSD test (*p* ≤ 0.05).

**Figure 2:**
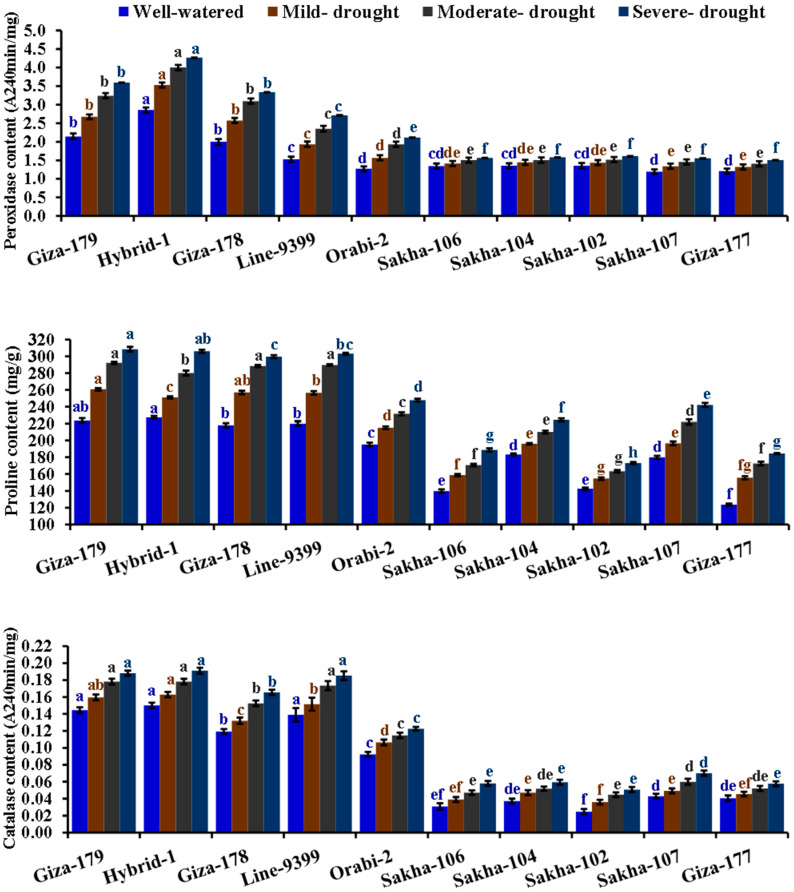
Impact of different irrigation regimes on the content of peroxidase, proline, and catalase of ten diverse rice genotypes over two growing seasons in 2019 and 2020. The top bars on each column indicate the standard error (SE). Distinct letters on columns of each irrigation regime (sharing the same color) reveal significant differences based on the LSD test (*p* ≤ 0.05).

The tested irrigation regimes significantly impacted all physiological parameters studied (Table 2). A gradual reduction was observed in the chlorophyll content and relative water content under mild, moderate, and severe drought conditions. While leaf rolling, the contents of peroxidase, proline, and catalase were increased. Under severe drought stress, chlorophyll and relative water content dropped by 27.3% and 21.1%, respectively, compared to well-watered conditions. Conversely, severe drought stress substantially increased leaf rolling, peroxidase, proline, and catalase contents by 205, 46.6, 33.7, and 40.2%, respectively, compared to well-watered conditions. There were significant differences among the evaluated genotypes for all physiological traits. Sakha 102, Giza 177, and Sakha 106 exhibited the highest values of leaf rolling. Moreover, the genotypes Hybrid-1, Giza 179, Line-9399, and Giza 178 showed superior content of chlorophyll, relative water, peroxidase, proline, and catalase. The interaction between assessed irrigation regimes and evaluated genotypes significantly affected the contents of chlorophyll, relative water, peroxidase, proline, and catalase. Notably, Giza 179, Hybrid 1, Giza 178, and Line 9399 recorded the greatest values for these parameters under drought stress conditions (Figure 1 and Figure 2).

### 3.2. Agronomic Traits and Crop Water Productivity

**Figure 3:**
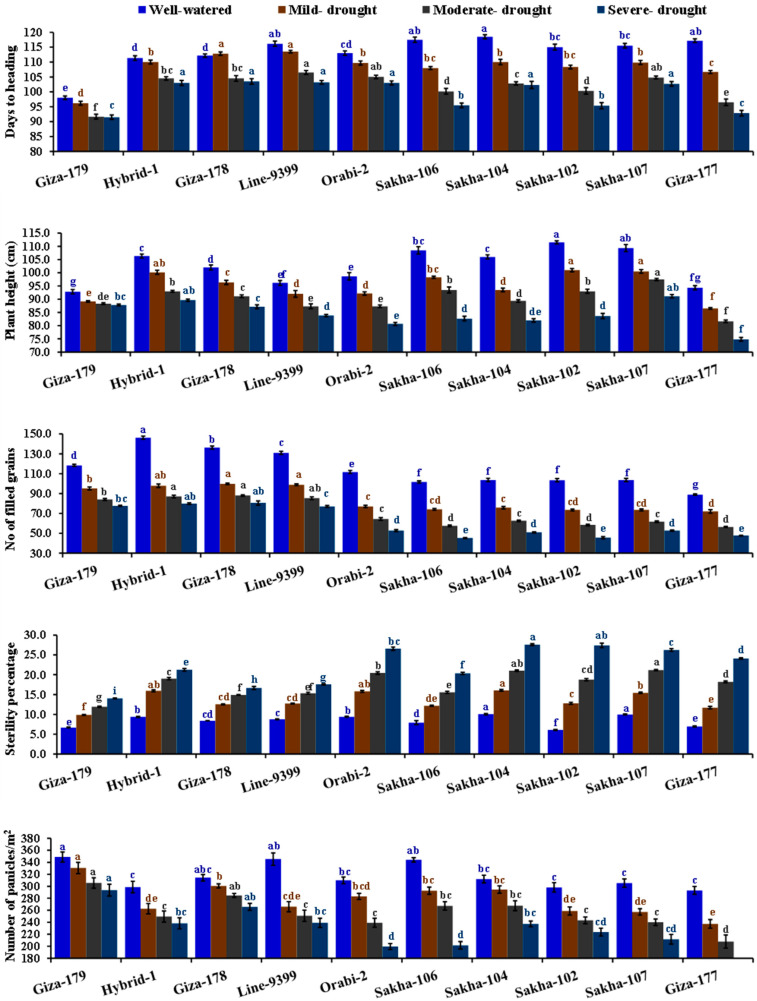
Impact of different irrigation regimes on days to heading, plant height, number of filled grains, sterility percentage, and number of panicles per square meter of ten diverse rice genotypes over two growing seasons in 2019 and 2020. The top bars on each column indicate the standard error (SE). Distinct letters on columns of each irrigation regime (sharing the same color) reveal significant differences based on the LSD test (*p* ≤ 0.05).

**Figure 4:**
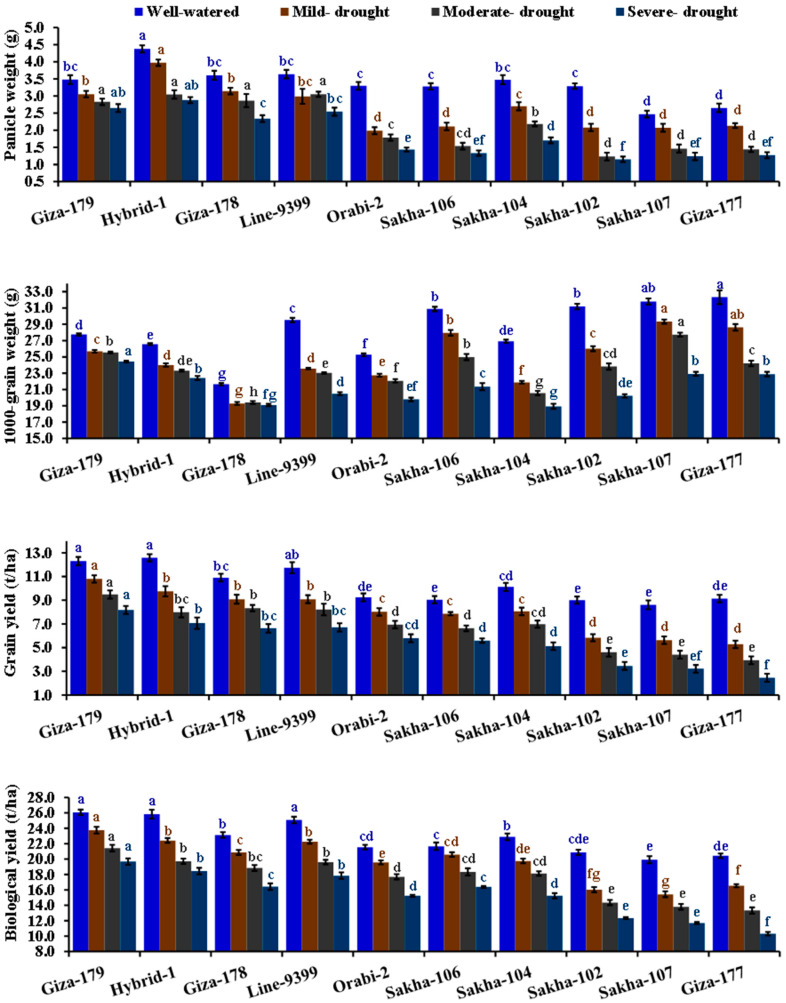
Impact of different irrigation regimes on panicle weight, 1000-grain weight, grain yield, and biological yield of ten different rice genotypes over two growing seasons in 2019 and 2020. The top bars on each column indicate the standard error (SE). Distinct letters on columns of each irrigation regime (sharing the same color) reveal significant differences based on the LSD test (*p* ≤ 0.05).

The irrigation regimes substantially influenced grain yield and its attributed traits (Table 3 and Table 4). Mild, moderate, and severe drought treatments displayed a gradual reduction in plant height, days to heading, number of filled grains, number of panicles/m^2^, 1000-grain weight, panicle weight, grain yield, and biological yield. Severe drought induced a notable decline in all traits as mentioned above by 17.8, 12.5, 46.7, 28.8, 25.1, 44.9, 47.2, and 32.4%, in the same order, compared to well-watered conditions. Conversely, severe drought stress significantly increased the sterility percentage by 165.3% compared to well-watered conditions. The genotypic differences were evident in grain yield and its related traits. The genotypes Sakha 107, Sakha 102, Hybrid 1, and Sakha 106 displayed the highest values of plant height. Additionally, Giza-179, Hybrid-1, Line-9399, and Giza-178 demonstrated superior values of days to heading, number of panicles per square meter, number of filled grains, grain yield, and biological yield, with the lowest values of sterility percentage (Table 4). Notably, the interaction between assessed irrigation regimes and evaluated genotypes significantly affected the number of panicles per square meter, number of filled grains, sterility percentage, plant height, panicle weight, thousand-grain weight, grain yield, and biological yield. The highest number of filled grains, number of panicles per square meter, panicle weight, grain yield, and biological yield were recorded by Hybrid-1, Giza-179, Line-9399, and Giza-178 under drought stress conditions (Figure 3 and Figure 4).

**Figure 5:**
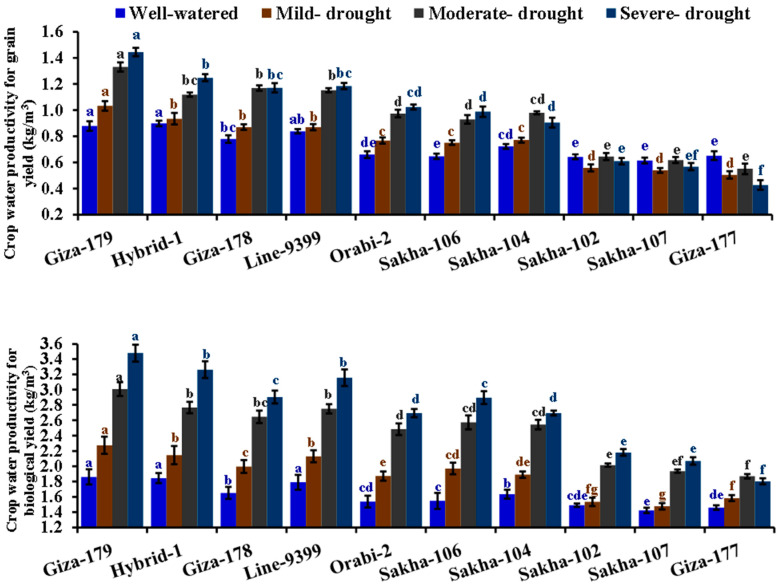
Impact of different irrigation regimes on crop water productivity for grain and biological yields of ten different rice genotypes over two growing seasons in 2019 and 2020. The top bars on each column indicate the standard error (SE). Distinct letters on columns of each irrigation regime (sharing the same color) reveal significant differences based on the LSD test (*p* ≤ 0.05).

The drought treatments caused a remarkable increase in crop water productivity for grain and biological yields. The most substantial crop water productivity was observed under severe drought conditions (Table 4). Under severe drought, crop water productivity for grain and biological yields increased by 30.4 and 67.02%, respectively, compared to well-watered conditions. Across the assessed genotypes, significant variations were observed in crop water productivity for grain and biological yields. Giza-179 exhibited the highest crop water productivity for grain yield, followed by Hybrid-1, Line-9399, Giza-178, Orabi-2, Sakha-104, and Sakha-106. d. The highest productivity was observed for biological yield in Giza-179, Hybrid-1, Line-9399, Giza-178, and Sakha-106. Moreover, the interaction between genotypes and irrigation regimes significantly influenced crop water productivity for both grain and biological yield. The highest crop water productivity for grain and biological yield was recorded by Giza-179, Hybrid-1, Giza-178, and Line-9399 under drought stress conditions (Figure 5).

### 3.3. Quality Characteristics

**Figure 6:**
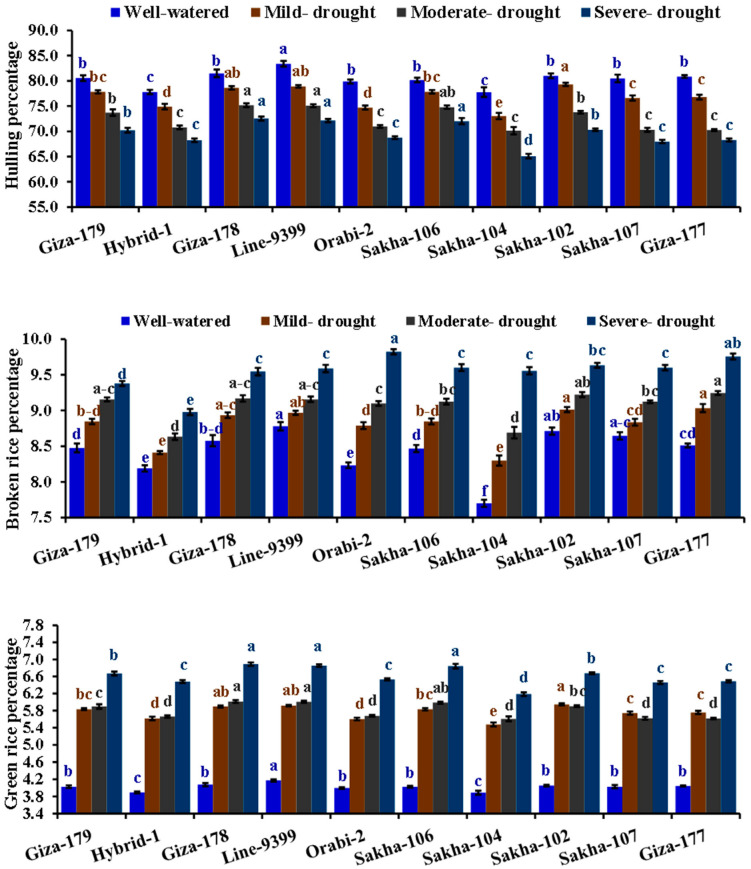
Impact of different irrigation regimes on hulling percentage, broken rice percentage, and green rice percentage of ten different rice genotypes over two growing seasons in 2019 and 2020. The top bars on each column indicate the standard error (SE). Distinct letters on columns of each irrigation regime (sharing the same color) reveal significant differences based on the LSD test (*p* ≤ 0.05).

The irrigation regimes significantly impacted all grain quality parameters (Table 5). Drought regimes gradually decreased the milling percentage, hulling percentage, and head rice percentage while increasing the broken and green rice percentages. Severe drought significantly reduced the milling, hulling, and head rice percentages by 13.3, 13.4, and 16.6% compared to well-watered conditions in the same order. Otherwise, severe drought stress significantly increased broken and green rice percentages by 13.3 and 64.4% compared to well-watered conditions, respectively. The assessed genotypes displayed a significant difference for all grain quality characteristics. The genotypes Line 9399, Giza 178, Giza 179, and Sakha 106 recorded superior percentages of hulling, milling and head rice. Furthermore, the lowest percentages of broken and green rice were assigned for Hybrid 1, Giza 179, and Sakha 104. The interaction between assessed irrigation regimes and evaluated genotypes was significant for hulling, broken, and green rice percentages. The highest hulling percentage and lowest percentages of broken and green rice were recorded by Giza 179, Line 9399, Giza 178, and Sakha 106 under drought stress conditions (Figure 6).

### 3.4. Genotypic Classification

**Figure 7:**
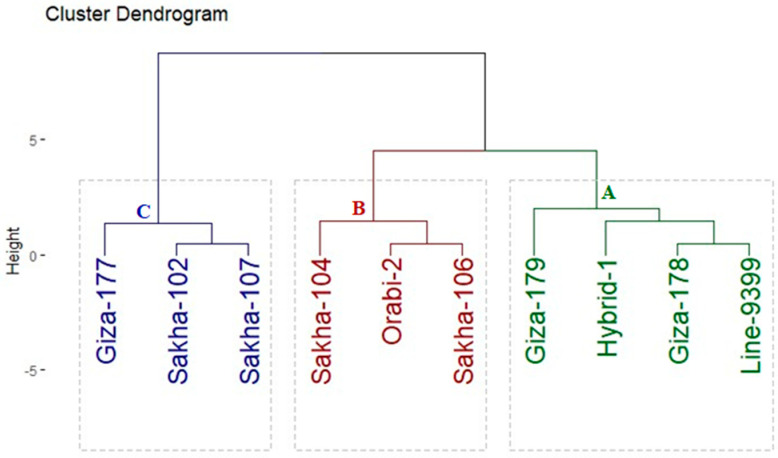
Dendrogram illustrating the distances among assessed rice genotypes based on drought tolerance indices. The genotypes were classified into three groups: A is drought tolerant (4 genotypes), B is moderately tolerant (3 genotypes), and C is sensitive (3 genotypes).

Four drought tolerance indices were computed based on the grain yield of each genotype under severe drought and well-watered conditions. These employed indices facilitated the classification of genotypes into three distinct groups utilizing hierarchical clustering (Figure 7). Group A contained four genotypes (Giza-179, Hybrid-1, Giza-178, and Line-9399) exhibiting the highest grain yield and tolerance indices, signifying its classification as a tolerant genotype. Likewise, Group B included three genotypes (Sakha-104, Orabi-2, and Sakha-106), displaying moderate tolerance index values, and thus classified as moderate drought-tolerant genotypes. In contrast, Group C comprised three genotypes (Giza-177, Sakha-102, and Sakha-107), exhibiting the lowest values in both grain yield and tolerance indices, which were consequently categorized as sensitive genotypes.

**Figure 8:**
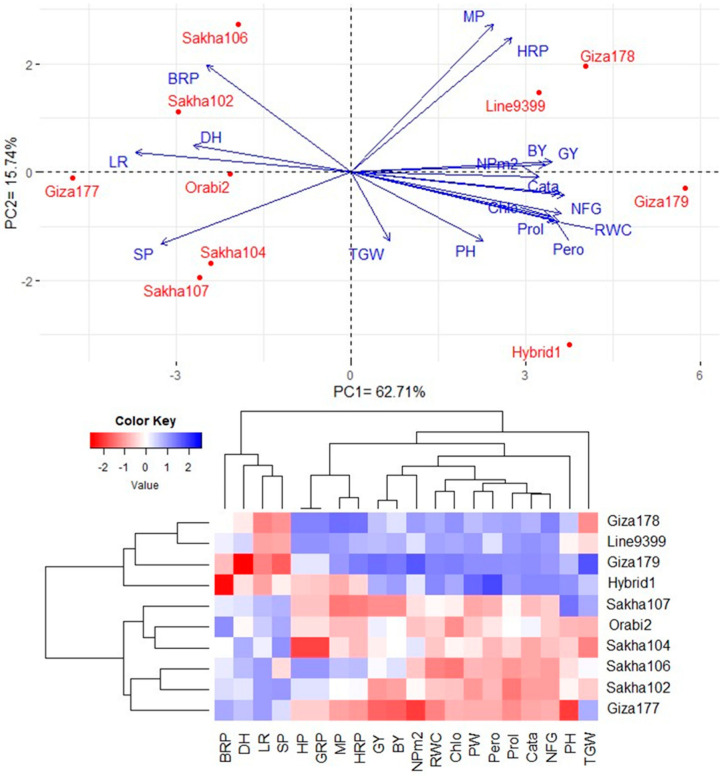
PC biplot and heatmap for assessed rice genotypes under drought stress based on physiological, agronomic, and grain quality over two growing seasons. The blue color reveals high values, while the red color indicates low values for the corresponding traits. LR: leaf rolling, RWC: relative water content, Chlo: chlorophyll content, Pero: peroxidase content, Prol: proline content, Cata: catalase content, PH: plant height, DH: days to heading, NFG: number of filled grains, NPm2: number of panicles per square meter, SP: sterility percentage, PW: TGW: 1000-grain weight, panicle weight, GY: grain yield, BY: biological yield, HP: hulling percentage, MP: milling percentage, HRP: head rice percentage, BRP: broken rice percentage, GRB: green rice percentage.

The first two principal components demonstrated approximately 78.45% of the variability (62.71% by PC1 and 15.74% by PC2). PC1 was associated with assessed genotypes, positioning high-yielding and tolerant genotypes (Giza-179, Hybrid-1, Giza-178, and Line-9399) towards the positive side (Figure 8). Conversely, low-yielding and sensitive genotypes (Giza-177) were located at the extreme opposing end of PC1. Tolerant genotypes exhibited positive associations with agronomic traits and most physiological and quality parameters. Meanwhile, the aforementioned traits showed negative associations with sensitive genotypes. Most studied parameters, except for broken rice percentage, leaf rolling, days to heading, and sterility percentage, demonstrated a strong positive relationship with grain yield. Based on the physiological, agronomic, and quality parameters studied, the heatmap grouped the assessed genotypes under drought stress into distinct clusters (Figure 8). Tolerant genotypes exhibited the highest values (depicted in blue), while sensitive genotypes showed the lowest values for most parameters studied (displayed in red).

## 4. Discussion

Climate change intensifies water scarcity, particularly in the arid regions of the Mediterranean, by increasing the severity, frequency, and duration of drought conditions [33,34]. These stresses pose a formidable challenge to rice productivity, directly threatening food security. Therefore, adopting water-conserving approaches becomes essential to both preserving water resources and ensuring the continued production of rice. Aerobic rice cultivation is a promising solution, significantly reducing water demand and enhancing water efficiency. Concurrently, cultivating drought-tolerant rice genotypes with high yields and grain quality is critical for sustainable rice production amidst sudden climatic shifts. The present study aimed to assess diverse rice genotypes under varied irrigation regimes to explore their physiological, agronomic, and grain quality responses under aerobic cultivation conditions. The results obtained showed a gradual decrease in the traits studied due to drought treatments (mild, moderate, and severe drought), highlighting the significant influence of drought stress on rice plant development and productivity. Physiological parameters such as chlorophyll content and relative water content were adversely affected by drought stress, which was reflected directly in yield traits and grain quality. The observed deleterious effect on yield traits and quality characteristics under drought stress might be attributed to decreased nutrient uptake, restricted nutrient transport from roots to shoots, and compromised membrane permeability [35]. In this respect, [36], Radha et al. [37] and Salgotra and Chauhan [38] elucidated the negative impacts of water scarcity on physiological, agronomic, and grain quality traits under drought stress. On the other hand, drought stress led to increased levels of peroxidase, proline, and catalase content in rice plants. The accumulation of these osmoprotectants and enzymatic antioxidants acts as a coping mechanism triggered by the induced drought stress [39].

The assessed rice genotypes exhibited notable variation in their physiological, agronomic, and grain quality traits under the irrigation regimes tested. This variability signifies genetic diversity present within these genotypes, offering potential opportunities for improving rice productivity and quality under drought stress conditions. Likewise, Shankar et al. [40] and Bhattacharjee et al. [41] depicted significant variation among rice genotypes under water deficit conditions in their physiological, agronomic, and quality traits. The evaluated genotypes were grouped into three different clusters according to drought response indices. These employed indices facilitated the classification of genotypes into three distinct groups utilizing hierarchical clustering. Giza-179, Hybrid-1, Giza-178, and Line-9399 were categorized as tolerant genotypes. Meanwhile, Sakha-104, Orabi-2, and Sakha-106 were considered moderate tolerant genotypes, and Giza-177, Sakha-102, and Sakha-107 were sensitive genotypes. The aforementioned drought-tolerant genotypes displayed elevated levels of peroxidase, proline, and catalase content in rice plants under drought stress. The activities of these osmoprotectants and enzymatic antioxidants play a critical role in preserving tissue water status and preventing cellular damage caused by water deficit [39]. Accordingly, these genotypes recorded higher yield traits, grain quality, and crop water productivity with reduced water usage than drought-sensitive genotypes under water deficit conditions. This suggests that these genotypes potentially have superior water and nutrient uptake abilities, alongside higher stomatal conductance [42]. These factors could contribute to enhanced photosynthesis, which, in turn, positively reflects on plant growth, productivity, and grain quality [43]. As a result, these genotypes are strongly recommended for cultivation in water-scarce environments. Furthermore, their characteristics could be valuable in breeding programs to enhance drought tolerance in rice, particularly under aerobic cultivation conditions.

Exploring the interrelationships among physiological, agronomic, and quality traits can provide valuable insights for screening rice genotypes under drought stress in breeding programs. Biplot analyses using principal components, along with heatmap and hierarchical clustering, serve as an effective statistical tools to visualize these relationships among studied traits [44,45]. The findings revealed robust associations between specific physiological parameters, such as relative water content, chlorophyll content, peroxidase content, proline content, and catalase content, with both yield-related and grain quality traits under drought stress. These parameters offer valuable insights and could serve as rapid indicators for assessing drought tolerance in rice breeding programs in arid environments.

## 5. Conclusions

Drought treatments adversely affected physiological traits, resulting in diminished yield traits and grain quality. This highlights the significant influence of drought stress on rice plant development, productivity, and grain quality. The assessed rice genotypes exhibited substantial genetic variability and differential responses to water deficit conditions. The genotypes Giza-179, Hybrid-1, Giza-178, and Line-9399 were classified as drought-tolerant and demonstrated higher yields and improved crop water productivity utilizing reduced water resources compared to sensitive genotypes. Accordingly, these genotypes are highly recommended for cultivation in water-scarce environments. In addition, their characteristics could offer significant value in breeding programs to improve drought tolerance in rice, especially under aerobic cultivation conditions.

## Figures and Tables

**Table 1:** Name, pedigree, origin, type, and grain shape of the assessed rice genotypes in the current study.

No.	Name	Pedigree	Origin	Type	Grain Shape
1	Giza-179	GZ62961XGZ1368-5-5-4	Egypt	Indica	Fine grain
2	Hybrid-1	IR696525AXGiza-178-R -	Egypt	Indica	Fine grain
3	Giza-178	(Giza-175/Milyang-49)	Egypt	Indica/Japonica	Medium grain
4	Line-9399	(GZ62961XGZ1368-5-5-4/Giza-175)	Egypt	Indica/Japonica	Medium grain
5	Orabi-2	A selected line from IR 47861321, after treating by EMS at 0.5%	Egypt	Indica	Fine grain
6	Sakha-106	Giza- 177XHexi30	Egypt	Indica	Fine grain
7	Sakha-104	(GZ-4096-8-1/GZ4100-9-1)	Egypt	Japonica	Short grain
8	Sakha-102	(GZ-4096-7-1/Giza-177)	Egypt	Japonica	Short grain
9	Sakha-107	GZ5581-46-3XGZGZ4316-7-1	Egypt	Japonica	Short grain
10	Giza-177	(Giza-171/Yomji No. 1//Pi No. 4)	Egypt	Japonica	Short grain

**Table 2:** Impact of different irrigation treatments on physiological characteristics of diverse rice genotypes over two seasons in 2019 and 2020.

Studied Factor		Leaf Rolling	Chlorophyll Content (SPAD)	Relative Water Content (%)	Peroxidase Content (A240 min/mg)	Proline Content (mg/g)	Catalase Content (A240 min/mg)
Irrigation regimes (IR)							
Well-watered		1.40 d	41.93 a	89.98 a	1.63 d	185.36 d	0.082 d
Mild drought		2.20 c	37.15 b	83.45 b	1.92 c	210.24 c	0.093 c
Moderate drought		3.42 b	33.90 c	77.20 c	2.20 b	232.15 b	0.105 b
Severe drought		4.27 a	30.47 d	70.98 d	2.39 a	247.83 a	0.115 a
Rice genotype (RG)							
Giza-179		0.71 c	41.90 a	93.74 a	2.92 b	271.27 a	0.168 b
Hybrid-1		1.25 c	40.53 b	91.68 b	3.66 a	266.19 b	0.171 a
Giza-178		0.83 c	40.19 b	89.99 c	2.75 c	265.80 b	0.142 d
Line-9399		1.17 c	39.56 c	91.71 b	2.13 d	267.35 b	0.162 c
Orabi-2		3.42 b	30.90 g	74.37 f	1.72 e	222.50 c	0.109 e
Sakha-106		4.83 a	29.67 h	67.62 g	1.46 f	164.58 f	0.044 h
Sakha-104		3.08 b	35.07 d	76.37 e	1.47 f	203.55 e	0.049 g
Sakha-102		4.67 a	32.27 f	73.61 f	1.48 f	158.45 g	0.039 i
Sakha-107		3.50 b	35.10 d	78.00 d	1.39 g	210.20 d	0.056 f
Giza-177		4.75 a	33.41 e	67.01 g	1.36 g	159.08 g	0.049 g
ANOVA	df		*p*-value				
Irrigation regimes (IR)	3	<0. 001	<0.001	<0.001	<0.001	<0.001	<0.001
Rice genotype (RG)	9	<0.001	<0.001	<0.001	<0.001	<0.001	<0.001
IR × RG	27	0.035	<0.001	<0.001	<0.001	<0.001	<0.001

The averages represent the main effect of the analyzed factors (irrigation regimes and rice genotypes). Means labeled with different letters within the same factor were significantly different based on the LSD test (*p* ≤ 0.05).

**Table 3:** Impact of different irrigation regimes on yield-attributing traits of diverse rice genotypes over two seasons in 2019 and 2020.

Studied Factor		Days to Heading	Plant Height (cm)	No. of Filled Grains	Sterility Percentage	No of Panicles/m^2^
Irrigation regimes (IR)						
Well-watered		113.43 a	102.57 a	114.47 a	8.36 d	316.98 a
Mild drought		108.50 b	94.97 b	83.76 b	13.50 c	278.35 b
Moderate drought		101.68 c	90.22 c	70.59 c	17.65 b	255.58 c
Severe drought		99.28 d	84.35 d	61.05 d	22.18 a	225.80 d
Rice genotype (RG)						
Giza-179		94.33 f	89.54 f	93.80 c	10.64 h	319.63 a
Hybrid-1		107.21 c	97.29 b	102.76 a	16.40 c	262.25 de
Giza-178		108.25 b	94.17 d	101.12 a	13.14 g	291.38 b
Line-9399		109.83 a	89.83 f	98.03 b	13.61 f	275.29 cd
Orabi-2		107.67 bc	89.71 f	76.50 d	18.05 b	257.88 de
Sakha-106		105.29 d	95.75 c	69.71 f	14.00 e	276.42 bc
Sakha-104		108.42 b	92.71 e	73.29 e	18.68 a	277.92 bc
Sakha-102		104.75 d	97.29 b	70.26 f	16.26 c	255.96 e
Sakha-107		108.21 b	99.63 a	72.93 e	18.21 b	253.54 e
Giza-177		103.29 e	84.33 g	66.29 g	15.24 d	221.54 f
ANOVA	df	*p*-value				
Irrigation regimes (IR)	3	<0.001	<0.001	<0.001	<0.001	<0.001
Rice genotype (RG)	9	<0.001	<0.001	<0.001	<0.001	<0.001
IR × RG	27	<0.001	<0.001	<0.001	<0.001	<0.001

The averages represent the main effects of analyzed factors (irrigation regimes and rice genotypes). Means labeled with different letters within the same factor were significantly different based on the LSD test (*p* ≤ 0.05).

**Table 4:** Impact of different irrigation regimes on panicle weight, 1000-grain weight (g), grain yield, biological yield crop water productivity of grain yield, and biological yield of diverse rice genotypes over two seasons in 2019 and 2020.

Studied Factor		Panicle Weight (g)	1000-Grain Weight (g)	Grain Yield (Ton/ha)	Biological Yield (Ton/ha)	Crop Water Productivity for Grain Yield (kg/m^3^)	Crop Water Productivity for Biological Yield (kg/m^3^)
Irrigation regimes (IR)							
Well-watered		3.36 a	28.39 a	10.27 a	22.75 a	0.734 d	1.625 d
Mild drought		2.63 b	24.91 b	7.94 b	19.73 b	0.760 c	1.888 c
Moderate drought		2.14 c	23.47 c	6.75 c	17.53 c	0.947 b	2.460 b
Severe drought		1.85 d	21.25 d	5.42 d	15.37 d	0.957 a	2.714 a
Rice genotype (RG)							
Giza-179		3.00 b	25.85 d	10.19 a	22.74 a	1.172 a	2.656 a
Hybrid-1		3.57 a	24.07 f	9.35 b	21.61 b	1.050 b	2.505 b
Giza-178		2.99 b	19.86 i	8.74 c	19.82 c	0.998 c	2.300 c
Line-9399		3.06 b	24.15 f	8.94 bc	21.20 b	1.012 c	2.458 b
Orabi-2		2.13 d	22.47 g	7.50 d	18.51 e	0.857 d	2.147 e
Sakha-106		2.06 de	26.29 c	7.28 d	19.25 d	0.829 d	2.247 cd
Sakha-104		2.52 c	22.08 h	7.57 d	19.01 d	0.845 d	2.192 de
Sakha-102		1.94 ef	25.31 e	5.72 e	15.90 f	0.614 e	1.806 f
Sakha-107		1.81 f	27.95 a	5.47 ef	15.22 g	0.585 e	1.727 fg
Giza-177		1.88 f	27.01 b	5.20 f	15.16 g	0.534 f	1.680 g
ANOVA	df	*p*-value					
Irrigation regimes (IR)	3	<0. 001	<0.001	<0.001	<0.001	>0.001	<0.001
Rice genotype (RG)	9	<0.001	<0.001	<0. 001	<0.001	<0.001	<0.001
IR × RG	27	<0.001	<0.001	<0.001	<0.001	<0.001	<0.001

The averages represent the main effect of analyzed factors (irrigation regimes and rice genotypes). Means labeled with different letters within the same factor were significantly different based on the LSD test (*p* ≤ 0.05).

**Table 5:** Impact of different irrigation regimes on grain milling quality parameters of diverse rice genotypes over two seasons in 2019 and 2020.

Studied Factor		Hulling Percentage	Milling Percentage	Head Rice Percentage	Broken Rice Percentage	Green Rice Percentage
Irrigation regimes (IR)						
Well-watered		80.34 a	69.23 a	60.48 a	8.43 d	4.02 c
Mild drought		76.85 b	65.55 b	56.62 b	8.80 c	5.76 b
Moderate drought		72.50 c	62.11 c	53.00 c	9.06 b	5.80 b
Severe drought		69.56 d	60.01 d	50.45 d	9.55 a	6.61 a
Rice genotype (RG)						
Giza-179		75.58 c	65.38 bc	56.30 ab	8.96 e	5.61 c
Hybrid-1		72.93 e	62.48 e	53.73 d	8.55 f	5.41 e
Giza-178		76.95 a	66.11 a	56.78 a	9.06 bc	5.72.a
Line-9399		77.38 a	65.88 ab	56.65 a	9.12 ab	5.74 a
Orabi-2		73.58 d	62.99 de	53.90 cd	8.99 de	5.46 d
Sakha-106		76.21 b	65.10 c	56.06 b	9.01 cde	5.67 b
Sakha-104		71.51 f	63.14 d	54.26 c	8.56 f	5.29 f
Sakha-102		76.10 bc	65.04 c	55.86 b	9.14 a	5.64 bc
Sakha-107		73.84 d	62.79 de	53.71 cd	9.05 cd	5.46 d
Giza-177		74.05 d	63.36 d	54.16 cd	9.14 a	5.48 d
ANOVA	df	*p*-value				
Irrigation regimes (IR)	3	<0.001	<0.001	<0.001	<0.001	<0.001
Rice genotype (RG)	9	<0.001	<0.001	<0.001	<0.001	<0.001
IR × RG	27	0.020	0.466	0.301	<0.001	<0.001

The averages represent the main effect of analyzed factors (irrigation regimes and rice genotypes). Means labeled with different letters within the same factor were significantly different based on the LSD test (*p* ≤ 0.05).

## Data Availability

The data presented in this study are available upon request from the corresponding author.

## Supplementary Materials

The following supporting information can be downloaded at: https://www.mdpi.com/article/10.3390/life14030370/s1. Table S1. Meteorological data for the two growing seasons of 2019 and 2020 at the experimental site. Table S2. Soil properties of the experimental site. Table S3. Scores and symptoms of leaf rolling at vegetative stage.
