# Supplementary material for: Physiological, Agronomic, and Grain Quality Responses of Diverse Rice Genotypes to Various Irrigation Regimes under Aerobic Cultivation Conditions
Source: Life (Basel). 2024 Mar 12;14(3):370. doi: 10.3390/life14030370 (PMC10972039; doi:10.3390/life14030370)
Supplement: Supplementary file 1 [file life-14-00370-s001.zip › life-2868245-supplementary.pdf]

Table S1. Meteorological data for the two growing seasons of 2019 and 2020 at the experimental site.

**Month**	**Temperature °C**		**Relative humidity%**	**Precipitation (mm)**
	**Maximum**	**Minimum**		
**Season 2019**				
May	28.50	11.60	84.70	0.00
June	31.70	17.00	86.20	0.00
July	31.30	17.50	84.40	0.00
August	33.00	18.60	91.30	0.00
September	33.00	16.50	88.30	0.00
October	29.00	13.40	89.40	0.00
**Season 2020**				
May	30.00	12.00	76.00	0.00
June	33.00	16.50	84.00	0.00
July	32.60	17.30	86.00	0.00
August	32.50	17.20	87.70	0.00
September	32.00	13.00	87.40	0.00
October	29.20	12.00	90.20	0.00

Table S2. Soil properties of the experimental site.

Character	Seasons			
	2019		2020	
	**0- 20***	**20- 40**	**0- 20**	**20- 40**
Chemical analysis				
PH	7.90	8.30	8.10	8.30
Organic matter (%)	1.50	1.65	1.40	1.50
Soluble cations (meq/l)				
Na^{+}	11.70	12.60	14.50	15.60
Ca^{++}	5.30	5.10	9.50	9.30
K^{+}	0.50	0.65	1.55	1.76
Mg^{++}	2.30	2.10	3.40	3.20
Soluble Anions (meq/l)				
Cl^{-}	9.90	9.10	8.10	8.40
$HCo3^{-}$	3.80	4.30	5.90	6.50
$So4^{--}$	13.50	17.50	14.25	14.80
Mechanical analysis				
Clay (%)	55.80	55.95	56.20	55.75
Sand (%)	13.45	13.85	12.70	13.60
Silt (%)	30.75	30.20	31.10	30.65
Soil texture	Clay	Clay	Clay	Clay

*Sample depth (cm)

Table S3. Scores and symptoms of leaf rolling at vegetative stage .

**Scores**	**Leaf rolling**
1	No rolling
3	Partially rolled, unrolled in the evening
5	Partially unrolling in late evening and early morning
7	Complete unrolling in the morning
9	Like tube no unrolling in the morning
